# What is the impact of national public expenditure and its allocation on neonatal and child mortality? A machine learning analysis

**DOI:** 10.1186/s12889-023-15683-y

**Published:** 2023-04-28

**Authors:** Leandro Pereira Garcia, Ione Jayce Ceola Schneider, Cesar de Oliveira, Eliane Traebert, Jefferson Traebert

**Affiliations:** 1 Graduate Program in Health Sciences, Universidade do Sul de Santa Catarina, Avenida Pedra Branca, 25, Palhoça, Santa Catarina 88132-260 Brazil; 2 Graduate Program in Rehabilitation Science, Public Health and Neuroscience, Universidade Federal de Santa Catarina, Rodovia Governador Jorge Lacerda, 3201, Araranguá, SC 88906-072 Brazil; 3 Department of Epidemiology and Public Health, University College London, 1-19 Torrington Place, London, WC1E 6BT UK; 4 School of Medicine, Universidade do Sul de Santa Catarina, Avenida Pedra Branca, 25, Palhoça, SC 88132-260 Brazil

**Keywords:** Child mortality, Neonatal mortality, Public expenditures, Cost allocation, Machine learning

## Abstract

**Background:**

Understanding the impact of national public expenditure and its allocation on child mortality may help governments move towards target 3.2 proposed in the 2030 Agenda. The objective of this study was to estimate the impacts of governmental expenditures, total, on health, and on other sectors, on neonatal mortality and mortality of children aged between 28 days and five years.

**Methods:**

This study has an ecological design with a population of 147 countries, with data between 2012 and 2019. Two steps were used: first, the Generalized Propensity Score of public spending was calculated; afterward, the Generalized Propensity Score was used to estimate the expenditures’ association with mortality rates. The primary outcomes were neonatal mortality rates (NeoRt) and mortality rates in children between 28 days and 5 years (NeoU5Rt).

**Results:**

The 1% variation in Int$ Purchasing Power Parity (Int$ PPP) per capita in total public expenditures, expenditure in health, and in other sectors were associated with a variation of -0.635 (95% CI -1.176, -0.095), -2.17 (95% CI -3.051, -1.289) -0.632 (95% CI -1.169, -0.095) in NeoRt, respectively The same variation in public expenditures in sectors other than health, was associates with a variation of -1.772 (95% CI -6.219, -1.459) on NeoU5Rt. The results regarding the impact of total and health public spending on NeoU5Rt were not consistent.

**Conclusion:**

Public investments impact mortality in children under 5 years of age. Likely, the allocation of expenditures between the health sector and the other social sectors will have different impacts on mortality between the NeoRt and the NeoU5Rt.

**Supplementary Information:**

The online version contains supplementary material available at 10.1186/s12889-023-15683-y.

## Background

More than five million children under five die annually [1]. Target 3.2 of the 2030 Agenda proposes to act on this issue, eliminating preventable deaths of newborns and children under five years of age [2]. Governments are responsible for implementing the 2030 Agenda and reducing child mortality. Unfortunately, there is no consensus on allocating public resources and the impact on infant mortality. Some studies, for example, found no relationship between investments in health and child mortality [3–5]. For example, a study in India, which accounts for more than 25% of global deaths, showed no relationship between public investments in health and mortality in children younger than one year [3]. However, other studies point to a relationship between public spending and mortality in this age group [6–17]. Deaths in children have many determinants, often interdependent. Most studies analyzing the relationship between these deaths and public investments use control strategies with limited determinants and a low capacity to deal with interdependencies. For lower-middle or low-income countries, data on many determinants are often missing and should be imputed for the analyses. Researchers https://ieeexplore.ieee.org/document/9923169, https://www.mdpi.com/2227-7390/10/8/1283  have used machine learning approaches to control for multicollinearity and nonlinear relationships in data. These techniques can be used in imputation and health impact evaluation, producing evidence to help governments achieve target 3.2 of the 2030 Agenda. So, in this study, a large amount of data on the determinants of child mortality has been collected over several years and in many countries. Appropriate techniques for imputation and control of determinants were used to produce such evidence.

The integrated and interdependent nature of the 2030 Agenda and the Sustainable Development Goals (SDGs) of the United Nations Development Program provides a powerful tool for global health organizations to support nations in addressing their determinants of health [18]. These goals can also be employed to fight child mortality. To do so, understanding its determinants is essential.

There are several determinants of neonatal deaths and deaths of children aged between 28 days and 5 years. The SDGs can be used as a framework for analyzing and identifying these determinants [18]. Income disparity in countries (SDG 10) and the Gross Domestic Product (GDP) (SDG 8), for example, have a strong relationship with infant deaths [19]. Poverty [20] (SDG 1) and schooling (mainly maternal) [21] (SDG 4 and SGD 5) rates are also related to infant mortality rates. Inadequate urbanization (SDG 11) and poor basic sanitation (SDG 6) are associated with diarrhea, which is responsible for 9% of deaths in this age group [1]. The scarcity of clean energy (SDG 7) for cooking, lighting, and indoor heating exposes women and children to pollutants generated by burning biofuels [22]. This exposure is associated with pneumonia, the leading cause of death (19%) among infectious diseases in children [1]. Public policies combined with a social protection system that promotes access to health services (SDG 3) for women and children, such as prenatal care, childbirth assisted by a qualified professional, and vaccination, are also important determinants of child mortality [23]. Similarly, health actions not explicitly associated with children, such as HIV [24] and malaria [1] control, can impact child mortality rates. Finally, child malnutrition (SDG 2) determines children’s vulnerability to the abovementioned circumstances [25]. Many of these deaths could be avoided by adequate social policies implemented by good public governance (SDG 16) [26]. Strong public governance can also enhance the impact of public spending (SDG 17) in this area [27].

Proper and responsible management of public spending can help reduce child mortality by impacting the network of determinants on several fronts. The way to organize these expenses can vary depending on the framework. There is a trade-off between public resources for the health sector and those for other government sectors. Therefore, as health expenditures and expenditures in other sectors comprise the national total expenses, the proportional increase in health investments will lead to a proportional decrease in different sectors and vice-versa. Furthermore, the impact of the determinants of health may differ by age group throughout childhood. So allocating resources between the health sector and other sectors can have a different impact on deaths at different ages [28]. A more comprehensive overview of the effects of ‘governments’ spending and the associated trade-offs in its allocation may help nations address child mortality.

Therefore, this investigation aimed to estimate the impacts of expenditure on the total national public budget, the health sector, and other government sectors on neonatal mortality (NeoRt) and mortality of children aged between 28 days and five years (NeoU5Rt).

## Methods

This study has an ecological design. Its sample population was comprised of countries with more than one million inhabitants. Countries with missing data on neonatal deaths, deaths in children under five, and the number of people by age group in the 2019 Global Burden of Disease databases (GBD) [29, 30] between 2018 and 2019 were excluded from the study. In addition, countries without data on general government spending, government spending on health, and foreign investment in health in the World Health Organization databases [31] between 2013 and 2017 have also been excluded.

**Fig. 1:**
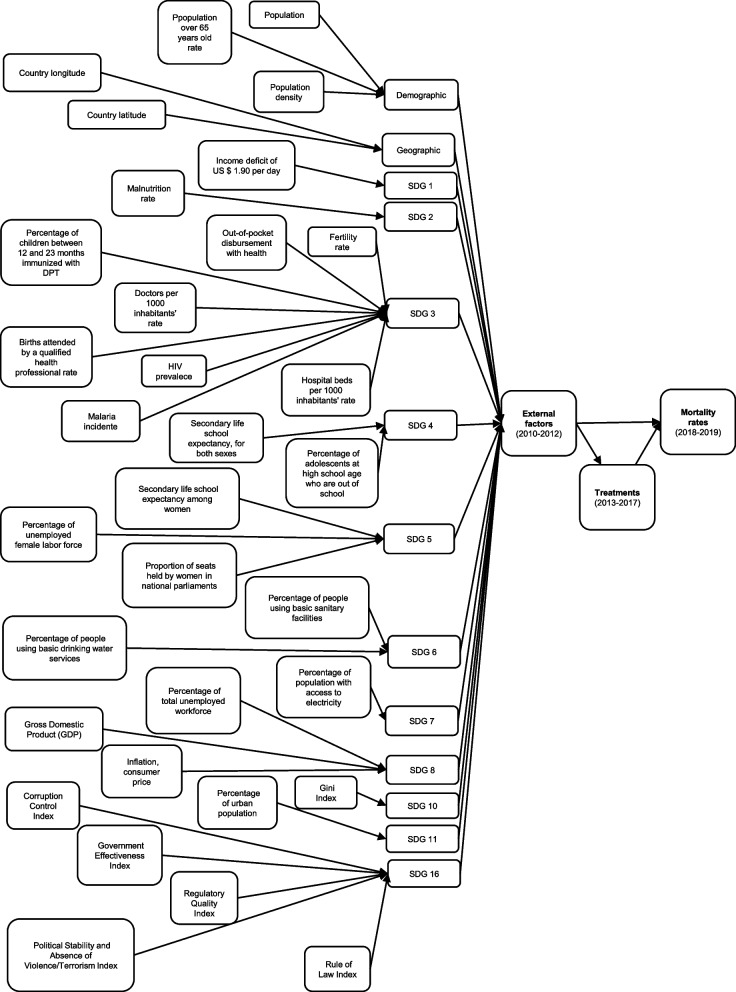
Logic model of analysis. Legend—The variables were grouped into three categories: mortality rates, treatments, or external factors. The mortality rates were the average neonatal mortality rate or average mortality rate of children aged 28 days to five years of age. The treatments were total public expenditure per capita, health public expenditure per capita or public expenditure in sectors other than health per capita. The external factors can impact both treatment and mortality rates. The thirty-three variables that form the external factors were organized using demographic, geographic, or Sustainable Development Goals structure as a framework. SDG—Sustainable Development Goals

The variables included in the analyses were organized into three groups: mortality, treatment, and external factors. The averages of NeoRt and NeoU5Rt composed the mortality group (outcome). The treatment group included total public expenditure per capita, public expenditure on health per capita, and public expenditure per capita with other sectors. The SDGs, the demographic, and the geographic characteristics served as a framework for the organization of 33 external factors, as shown in Fig. 1. Data on external factors were extracted from the World Bank database from 2010 to 2012 [32]. The complete list of data used in this survey and its source of extraction can be found in the Supplementary material (Table S1).

Variables in different periods were used to avoid bidirectional causation. More than one year was used for each variable category to increase the probability of completeness of the data. The external factors were calculated considering the years 2010 to 2012 averages. For the treatment variables, the average of the period 2013 to 2017 was used, and the period 2018 to 2019 was used for mortality. All financial values were extracted in Int$ Purchasing Power Parity (Int$ PPP), maintained constant for 2017 to allow comparison between countries and in different periods, and transformed into logarithms.

As previously mentioned, countries with missing data for calculating mortality (outcome) and treatment variables were excluded. Missing data were imputed to external factors to increase the participation of low- or lower-middle-income countries in the results because this group tends to have more missing data in the databases reviewed. Therefore, the Classification and Regression Tree method (CART) [33] was used for imputation. The CART algorithm, introduced by Breiman et al. [34] is a well-known class of machine learning techniques. It aims to identify potential predictors and cut-off points in those predictors to divide the sample into more homogenous subgroups. A binary tree can be constructed by recursively performing this splitting process on the resulting subgroups. This tree can be used to predict a target variable, which can be either discrete (in the case of a classification tree) or continuous (in the case of a regression tree). This method has been used with good results for variable imputation [35] and was implemented by the Multivariate Imputation by Chained Equations—MICE package [36] in the R language.

Variables with significant differences between imputed and observed data densities were excluded. Figure S1 of the Supplementary material shows the density graphs of the variables subject to imputation.

A two-step approach was used to control the factors influencing mortality and treatment simultaneously [37–39]. In the first stage, the Generalized Propensity Score (GPS) was estimated [40]. To that effect, the bivariate correlation of each of the external factors investigated with each of the treatments was analyzed. In the bivariate analysis, the Pearson correlation test was used. Only those external factors whose correlation had a *p*-value ≤ 0.25 were maintained [41]. Next, the result produced by three models to estimate the GPS was compared: SuperLearner [42], Propensity Score (PS) [43], and Covariate Balancing Propensity Score (CBPS) [44]. In the SuperLearner model, an assembly of algorithms was used [45–47] (generalized linear model [48], neural network [49], non-negative least squares [50], random forest [51], gradient boosting machine [52] and xgboost [53]). The propensity score is a vital tool in causal inference research. Rosenbaum and Rubin [54] showed that an unbiased estimate of the average treatment effect could be obtained by adjusting the propensity score alone. However, the propensity score’s accuracy is challenged by slight model misspecifications, resulting in a substantial bias of estimated treatment effects. The covariate balancing propensity score (CBPS) mitigates this by optimizing covariate balance and incorporating standard estimation procedures. CBPS can be extended to other causal inference settings, inherits all theoretical properties in the GMM literature, and allows for implementing various propensity score methods without modification [44]. Machine learning methods provide an alternative nonparametric approach to propensity score estimation. SuperLearner was proposed to choose the optimal machine learning regression algorithm among a set of candidates. Studies [45, 47] suggest that using SuperLearner to estimate the propensity score can improve covariate balance and reduce bias in cases of significant model misspecification for treatment assignment. Thus, SuperLearner was used with more conventional methods, such as PS and CBPS, due to the large number of multicollinear variables used for confounding control. Tree-based algorithms, such as random forest, gbm, and xgboost, can deal with this issue. Furthermore, the nonparametric approach of these algorithms can overcome difficulties with the distribution of such variables [55]. Weights were truncated between 10 and 90% to avoid outliers. The model with the smallest coefficient of variation of the weights, given by the standard deviation divided by the average of the weights [56], was selected for the impact analysis.

In the second stage, the bivariate analysis was repeated, but this time with the external factors group of variables with each one of the mortality variables. Finally, a least squares regression of the GPS treatment and external factors with *p* ≤ 0.25 [41] on the NeoRt and NeoU5Rt was performed. The final model was obtained by treating the external factors selected by backward step selection to produce the smallest Akaike Information Criteria (AIC) [57]. The 95% confidence intervals of the treatment ‘variables’ coefficients were calculated using the Delta method [58].

The analysis was repeated, excluding all imputed variables to assess the sensitivity to imputation. The sensitivity to the construction of the GPS was analyzed, testing the algorithm with the worst performance i.e., with the highest coefficient of variation of the weights.

All analyses were performed using the RStudio, version 1.1.463 [59] with version 3.5.3 R software [60]. Scripts and databases are publicly available at https://github.com/lpgarcia18/public_resource_impact_children_mortality.

## Results

**Fig. 2:**
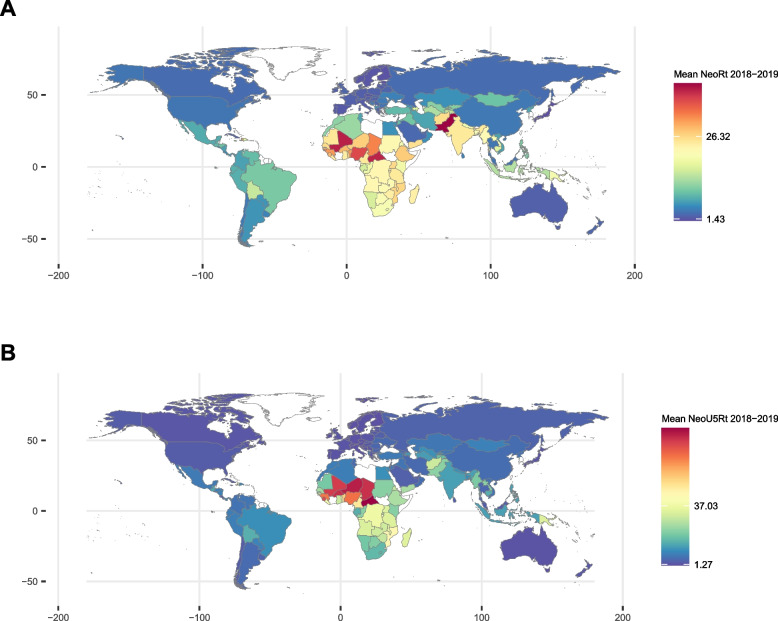
Distribution of mortality rates. Legend: **A** Average neonatal mortality rate of the years 2018 and 2019, **B** Average children aged 28 days to five years old mortality rate of the years 2018 and 2019. The values in the color legend in charts **A** and **B** indicate the 5^th^ and 95^th^ percentiles of each of the variables. Mean NeoRt 2018–2019: Average neonatal mortality rate of the years 2018 and 2019; Mean NeoU5Rt 2018–2019: Average children aged 28 days to five years old mortality rate of the years 2018 and 2019

**Fig. 3:**
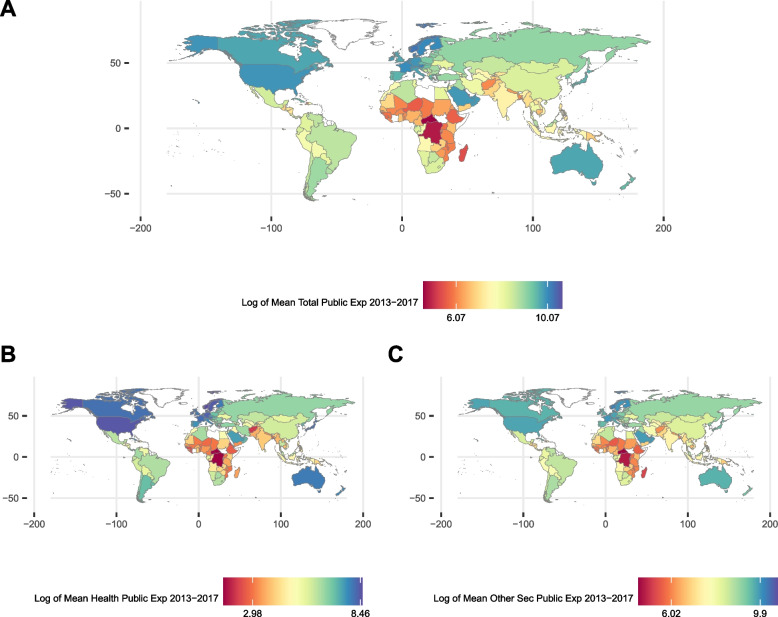
Distribution of treatments. Legend: **A** Natural logarithm of average public expenditure per capita of the years 2013 to 2017; **B** Natural logarithm of average health public expenditure per capita of the years 2013 to 2017; **C **Natural logarithm of average public expenditure in other sectors than health per capita of the years 2013 to 2017. The values in the color legend in charts **A**, **B**, and **C** indicate the 5^th^ and 95^th^ percentiles of each of the variables. Log of Mean Total Public Exp 2013–2017: Natural logarithm of average public expenditure per capita of the years 2013 to 2017; Log of Mean Health Public Exp 2013–2017: Natural logarithm of average health public expenditure per capita of the years 2013 to 2017; Log of Mean Other Sec Public Exp 2013–2017: Natural logarithm of average public expenditure in other sectors than health per capita of the years 2013 to 2017

A total of 147 countries were included in the study. The country with the lowest NeoRt was Japan, with 0.88 deaths per 1,000 live births, and the country with the highest NeoRt was Pakistan, with 42.29 deaths per 1,000 live births. Slovenia had the lowest NeoU5Rt, 0.79 per 1,000 live births, and the Central African Republic, with the highest, 84.52 per 1,000 live births. The average per capita public expenditure in these countries was most elevated in Qatar (Int$ PPP 45,402.26) and lowest in the Central African Republic (Int$ PPP 102.30). As for the average public spending on health, Norway had the highest values (Int$ PPP 5,293.14) while the Democratic Republic of Congo had the lowest (Int$ PPP 4.53). The average amount spent on sectors other than health was highest in Qatar (Int$ PPP 42,553.47) and lowest in the Central African Republic (Int$ PPP 97.73). The distributions of mortality rates and treatment variables in the two groups of countries are shown in Figs. 2 and 3.

The following variables were imputed: Inflation, consumer price; Percentage of people using basic drinking water services; Secondary school life expectancy among women; Proportion of seats held by women in national parliaments; Secondary life school expectancy for both sexes; Births attended by a qualified health professional; Percentage of adolescents of high school age who are out of school; Malnutrition rate; HIV prevalence; Malaria incidence; Doctors per 1000 inhabitants’ rate; and Hospital beds per 1000 inhabitants’ rate and Percentage of population with access to electricity. Gini Index; Income deficit of US$ 1.90 were excluded. The impact evaluation used the imputed data and the model that produced the smallest variation coefficient in the weights, which was the CBPS. According to this assessment, the 1% increase in Int$ PPP per capita of total public spending, health sector spending, and spending on other sectors was associated with a reduction of -0.635 (95% CI -1.176, -0.095), -2.17 (95% CI -3.051, -1.289) -0.632 (95% CI -1.169, -0.095) in NeoRt, respectively. The sensitivity analysis of the imputation of data was done excluding all imputed variables. This analysis indicated that the same amount of increase in total expenditure, health expenditure, and expenditure on other sectors was associated with a reduction of -2.559 (95% CI -3.633, -1.486), -2.438 (95%CI -3.286, -1.589) and -2.478 (95% CI -3.602, -1.354) in NeoRt, respectively. The sensitivity analysis of the GPS modelusing SuperLearner, the model that produced the highest weight variation coefficients in all analyses, indicated that an increase in total expenditure, health expenditure, and expenditure on other sectors was associated with a reduction of -7.247 (95% CI -11.504, -2.99), -2.445 (95% CI -3.299, -1.591) and -2.117 (95% CI -3.405, -0.829) in NeoRt, respectively. Therefore, both the impact analysis and the sensitivity analyses pointed to an association between an association of increase in total, in heath, and in other sectors of public spending with a reduction in NeoRt (Table 1).

**Table 1:** Impact of public expenditures on mortality rates

**Treatment**	**Coefficient** **(CI 95%)** **(CI 99%)** **(CI 99.9%)**					
	**Mean NeoRt 2018–2019**			**Mean NeoU5Rt 2018–2019**		
	Impact analysis	Sensitivity analysis 1	Sensitivity analysis 2	Impact analysis	Sensitivity analysis 1	Sensitivity analysis 2
Log of Mean Total Public Exp 2013–2017	-0.635 (-1.176, -0.095) (-1.35, 0.079) (-1.555, 0.284)	-2.559 (-3.633, -1.486) (-3.978, -1.141) (-4.385, -0.734)	-7.247 (-11.504, -2.99) (-12.874, -1.621) (-14.497, 0.002)	-1.728 (-3.283, -0.173) (-3.783, 0.326) (-4.374, 0.917)	-4.326 (-7.085, -1.567) (-7.971, -0.681) (-9.019, 0.367)	-0.287 (-0.681, 0.108) (-0.808, 0.235) (-0.958, 0.384)
Log of Mean Health Public Exp 2013–2017	-2.17 (-3.051, -1.289) (-3.334, -1.006) (-3.67, -0.67)	-2.438 (-3.286, -1.589) (-3.558, -1.317) (-3.88, -0.995)	-2.445 (-3.299, -1.591) (-3.573, -1.316) (-3.898, -0.991)	-2.17 (-3.051, -1.289) (-1.318, 6.143) (-2.392, 7.217)	5.842 (3.375, 8.31) (2.583, 9.101) (1.648, 10.037)	-0.439 (-0.927, 0.049) (-1.083, 0.206) (-1.269, 0.392)
Log of Mean Other Sec Public Exp 2013–2017	-0.632 (-1.169, -0.095) (-1.341, 0.078) (-1.546, 0.282)	-2.478 (-3.602, -1.354) (-3.962, -0.994) (-4.389, -0.568)	-2.117 (-3.405, -0.829) (-3.819, -0.415) (-4.309, 0.076)	-1.772 (-3.216, -0.329) (-3.679, 0.134) (-4.228, 0.683)	-3.839 (-6.219, -1.459) (-6.983, -0.695) (-7.886, 0.208)	-1.49 (-2.752, -0.227) (-3.157, 0.178) (-3.637, 0.658)

*Legend:* *CI 95%:* 95% Confidence Interval; Mean NeoRt 2018–2019: Average neonatal mortality rate of the years 2018 and 2019; Mean NeoU5Rt 2018–2019: Average children aged 28 days to five years old mortality rate of the years 2018 and 2019; Log of Mean Total Public Exp 2013–2017: Natural logarithm of average public expenditure per capita of the years 2013 to 2017; Log of Mean Health Public Exp 2013–2017: Natural logarithm of average health public expenditure per capita of the years 2013 to 2017; Log of Mean Other Sec Public Exp 2013–2017: Natural logarithm of average public expenditure in other sectors than health per capita of the years 2013 to 2017; Impact analysis: analysis with imputed data and using the model that produces the smallest coefficient of variation on weights; Sensitivity analysis 1: analysis without imputed data; Sensitivity analysis 2: analysis using the model that produces the largest coefficient of variation on weights

The 1% increase in Int$ PPP per capita of the total public expenditure, expenditure on the health sector, and expenditure on sectors other than health were associated with -1.728 (95% CI -3.283, -0.173), -2.17 (95% CI -3.051, -1.289) and -1.772 (95% CI -3.216, -0.329) reduction in NeoU5Rt, respectively. The data imputation sensitivity analysis also indicated a negative association between the increase in total public expenditure [-4.326 (95% CI -7.085, -1.567)] and spending on sectors other than health [-3.839 (95% CI -6.219, -1.459))] in NeoU5Rt reduction. In contrast, the increase in public health spending was associated with an increase in this rate [1.80 (95% CI -1.21, 4.80)]. The sensitivity analysis of the GPS model showed the following results for the rise in expenditure on sectors other than health: –1.49 (95% CI -2.752, -0.227). Both the total expenditure and the health expenditure were not associated with NeoU5Rt d in sensitivity analyses of the GPS (Table 1).

## Discussion

Key findings from this study point to an association between increased total public spending, spending on health, and spending on other sectors and reduced NeoRt. Likewise, an association between the rise in expenditure on other sectors and a decrease in NeoU5Rt. Among the countries assessed, a 1% increase in total public spending was associated with a reduction of around 0.64 deaths in the neonatal period for every 1,000 live births. These findings align with other studies [3, 6–10, 61] that showed public investment as protection against child mortality.

Evidence in this study raises the hypothesis that the allocation of public funds may have different effects on mortality among children in the neonatal period and in older children. It meets the pathophysiological mechanisms for deaths in children in the neonatal period and older children. In the neonatal period, for example, health actions such as adequate prenatal care and childbirth assisted by a qualified professional are essential for reducing mortality [23]. In turn, adequate antenatal care and skilled birth attendance can be influenced by female education [62]. Thus, investment in education is related to health care that impacts NeoRt. For older children, who are already at home, the lack of basic sanitation [1], the lack of clean energy for cooking, lighting, and heating environments [22], and external causes [63], for example, are major determinants of mortality. Previous evidence has shown complex interactions, some synergistic [64–70] and others deleterious [64, 71], among the SDGs, here addressed from the perspective of the social determinants of health. Therefore, the differences observed can be explained by the different weights of these determinants in each age group during childhood. In Bangladesh [28], for example, the odds ratio (OR) of neonatal death among children of mothers with tertiary education is 0.42 (95% CI 0.31, 0.55), compared to children of mothers with no education. For children under 5 years of age, the OR is 0.27 (95% CI 0.21, 0.34), indicating a greater importance of maternal education in protecting older children.

The different effects mentioned earlier may be a potential source of conflicting findings in the literature. Some studies showed the impact of health investment on child mortality [6–17] while sometimes failing to demonstrate it [3–5]. For example, Makela et al. [3], in a study in India, a country that concentrates 25% of child deaths globally, showed different impacts on children between one and four years of age between health investments and investments in other sectors. The authors found a relationship between health expenditure and mortality rate only for boys aged one to four years. However, increased spending on education, social sectors other than health, and a reduction in the poverty rate were consistently associated with a reduction in mortality in both genders [3]. An essential objective of the study was greater participation from low- and lower-middle-income countries. This participation is important in international studies on child mortality. Although the data on social determinants is large enough for several countries, there are more missing data in low- and middle-income countries, which concentrate a large number of these deaths. Other strengths of this analysis are the use of the GPS, the control of bidirectional causation, and the sensitivity analysis to reduce the impact of bias. Machine learning strategies, such as those employed in this study, have been used due to their flexibility in complex interaction modeling [72], such as those between SDGs [73]. However, despite this potential, CBPS was the model that produced the lowest variation coefficient of weights, and SuperLearner was the largest, showing the importance of testing several models to estimate the GPS.

Among the limitations of this study is using the general inflation rate to control the evolution of costs in the health and non-health sectors. Studies have indicated that inflation in the health sector is generally higher [74], but these data are not available for most of the analyzed countries. Another important point is that the distribution of missing data is not random. It is more prevalent in low-income countries than in others, which may lead to bias. We tried to control the possibility of bias through sensitivity analysis. The findings must also be generalized carefully since this study is ecological, and the effects may be heterogeneous. Future studies to analyze the heterogeneity of the relationship between the allocation of public resources and infant mortality can help to refine the current evidence and inform the decision-making of public managers.

## Conclusions

The present analysis groups countries, focusing on an average association across the countries assessed. This association likely varies according to the context of individual countries, preventing the interpretation from being generalized for a given country. Furthermore, social protection, sanitation, and education systems have developed over several years. An attempt was made to control the structure built by past public spending, limiting the analysis to public spending from 2013 to 2017. Therefore, the impact of the accumulation of longer-term investments was not analyzed. With a more extended time window, future research can better capture the effects of government structural investments.

The 2030 Agenda stresses the importance of achieving children’s health and well-being, in order not leaving anyone behind. Our key findings indicated that increases in total, health, and other sectors governments’ expenditure can reduce NeoRt. Investments in sectors other than health can decrease NeoU5Rt. Adequate resource allocation may help to advance toward goal 3.2 of the 2030 Agenda. However, allocating these investments between the health sector and other government sectors needs to be further investigated.

## Supplementary Information

**Additional file 1: Table S1.** Study variables description. **Table S2.** Study variables distribution. **Table S3.** Weights’ coefficients of variation. **Figure S1.** Legend: The density of the imputed data for each data set is in red and that of the observed data in blue. GINI_LAGGED: Gini Index; POVERTY_GAP_LAGGED: Income deficit of US$ 1.90; INFLATION_LAGGED: Inflation, consumer price ; BASIC_WATER_LAGGED: Percentage of people using basic drinking water services; SCHOOL_FEM_LAGGED: Secondary school life expectancy among women; WOMEN_PARLIAMENT_LAGED: Proportion of seats held by women in national parliaments; SCHOOL_LIFE_EXP_LAGGED: Secondary life school expectancy, for both sexes; OUT_OF_SCHOOL_LAGGED: Percentage of adolescents at high school age who are out of school; UNDERNOURISHMENT_LAGGED: Malnutrition rate; DOCTORS_LAGGED: Doctors per 1000 inhabitants’ rate; DELIVERY_ASSISTANCE_LAGGED: Births attended by a qualified health professional; AIDS_PREVALENCE_LAGGED: HIV prevalence; MALARIA_INCIDENCE_LAGGED: Malaria incidence; HOSPITAL_BEDS_LAGGED: Hospital beds per 1000 inhabitants’ rate; ELECTRICITY_LAGGED: Percentage of population with access to electricity.

## Data Availability

The datasets generated and/or analyzed during the current study are available in the https://github.com/lpgarcia18/public_resource_impact_children_mortality.

## Abbreviations

NeoRt: Neonatal mortality rates
NeoU5Rt: Mortality rate in children aged between 28 days and 5 years
Int$ PPP: Int$ Purchasing Power Parity
SDG: Sustainable Development Goal
GDP: Gross Domestic Product
GBD: Global Burden of Disease
CART: Classification and Regression Tree
GPS: Generalized Propensity Score
PS: Propensity Score
CBPS: Covariate Balancing Propensity Score
AIC: Akaike Information Criteria
