# Supplementary material for: What is the impact of national public expenditure and its allocation on neonatal and child mortality? A machine learning analysis
Source: BMC Public Health. 2023 Apr 28;23:793. doi: 10.1186/s12889-023-15683-y (PMC10141942; doi:10.1186/s12889-023-15683-y)
Supplement: Supplementary file 1 — Additional file 1: Table S1. Study variables description. Table S2. Study variables distribution. Table S3. Weights’ coefficients of variation. Figure S1. Legend: The density of the imputed data for each data set is in red and that of the observed data in blue. GINI_LAGGED: Gini Index; POVERTY_GAP_LAGGED: Income deficit of US$ 1.90; INFLATION_LAGGED: Inflation, consumer price ; BASIC_WATER_LAGGED: Percentage of people using basic drinking water services; SCHOOL_FEM_LAGGED: Secondary school life expectancy among women; WOMEN_PARLIAMENT_LAGED: Proportion of seats held by women in national parliaments; SCHOOL_LIFE_EXP_LAGGED: Secondary life school expectancy, for both sexes; OUT_OF_SCHOOL_LAGGED: Percentage of adolescents at high school age who are out of school; UNDERNOURISHMENT_LAGGED: Malnutrition rate; DOCTORS_LAGGED: Doctors per 1000 inhabitants’ rate; DELIVERY_ASSISTANCE_LAGGED: Births attended by a qualified health professional; AIDS_PREVALENCE_LAGGED: HIV prevalence; MALARIA_INCIDENCE_LAGGED: Malaria incidence; HOSPITAL_BEDS_LAGGED: Hospital beds per 1000 inhabitants’ rate; ELECTRICITY_LAGGED: Percentage of population with access to electricity. [file 12889_2023_15683_MOESM1_ESM.docx]

**SUPPLEMENTARY MATERIAL**

Table S1 – Study variables description.

| **Mortality – average of 2018 to 2019** | | **Description** |
| --- | --- | --- |
| Neonatal mortality rate | | The average neonatal mortality rates of the years 2018 and 2019 was calculated for each country. The number of deaths up to 28 days was divided by the number of live births and multiplied by 1000. Data were extracted from the Global Burden of Disease 2019. |
| Mortality rate among children aged 28 days or more and less than five years old | | Average mortality rate among children aged 28 days or more and less than five years old of the years 2018 and 2019 was calculated for each country. The number of deaths among children aged 28 days or more and less than five years old was divided by the number of live births and multiplied by 1000. Data were extracted from the Global Burden of Disease 2019. |
| **Treatment – average of 2013 to 2017** | | **Description** |
| Public expenditure per capita – Int$ PPP constant for 2017 | | Public expenditure per capita was the value of general government spending (in Int$ Purchasing Power Parity - Int$ PPP constant for 2017). Average public expenditure by country of the years 2013 and 2017 was calculated. |
| Public expenditure in health per capita – Int$ PPP constant for 2017 | | Public expenditure in health per capita was the value of domestic general government health expenditure (Int$ PPP constant for 2017). Average public expenditure by country of the years 2013 and 2017 was calculated. |
| Public expenditure in other sectors per capita – Int$ PPP constant for 2017 | | Public expenditure in other sectors per capita was the difference between public expenditure per capita and public expenditure in health per capita. |
| **External factor – average of 2010 to 2012** | **Demography, geography or SDG** | **Description** |
| Population | Demography | Average of the country's total population from 2010 to 2012. |
| Population density | Demography | Population density was calculated by dividing the average of the country's total population from 2010 to 2012, by its surface. Surface is the total area of a country, including areas under inland bodies of water and some coastal roads [2]. This information was extracted in km^2^ by country. |
| Population over 65 years old rate | Demography | The average population over 65 years old rate was estimated dividing the population over 65 years old by the population of a country in a given year and making the average for 2010 to 2012. |
| Countries longitude | Geography | The geometric center of each country was determined using the WGS84 datum, using the data available in spData [3] and using the sf^2^ package, from software R, for the calculation. |
| Country latitude | Geography | The geometric center of each country was determined using the WGS84 datum, using the data available in spData [3] and using the sf^2^ package, from software R, for the calculation. |
| Income deficit of US$ 1.90 | SDG 1 | The US$ 1.90 per day income deficit (2011 Int$ PPP) is the average income or consumption deficit from the US$ 1.90 per day poverty line. This measure reflects the depth of poverty, as well as its incidence [4]. The average of the years 2010 to 2012 was calculated for each country. |
| Malnutrition rate | SDG 2 | The population below the minimum level of energy consumption in the diet (also known as malnutrition prevalence) shows the percentage of the population whose food intake is insufficient to continuously meet the energy needs of the diet [5]. The average for the years 2010 to 2012 was calculated for each country. |
| Fertility rate | SDG 3 | The average fertility rate was calculated using total fertility data for each country from 2010 to 2012. |
| Out-of-pocket disbursement with health per capita – Int$ PPP constant for 2017 | SDG 3 | Out-of-pocket disbursement with health per capita in Int$ PPP constant for 2017. The average for the years 2010 to 2012 was calculated, for each country. |
| Doctors per 1000 inhabitants’ rate | SDG 3 | Doctors include general practitioners and specialists. The value is divided by 1000 inhabitants [6]. The average from 2010 to 2012 was calculated for each country. |
| Births attended by a qualified health professional | SDG 3 | Births attended by qualified health personnel are the percentage of births attended by personnel trained to provide the necessary supervision, care and advice to women during pregnancy, labor and the postpartum period; deliver on your own; and taking care of newborns [7]. The average from 2010 to 2012 was calculated for each country. |
| HIV prevalence | SDG 3 | HIV prevalence refers to the percentage of people aged 15 to 49 who are infected with HIV [8]. The average from 2010 to 2012 was calculated for each country. |
| Malaria incidence | SDG 3 | Malaria incidence is the number of new cases of malaria in one year per 1000 inhabitants at risk [9]. The average from 2010 to 2012 was calculated for each country. |
| Percentage of children between 12 and 23 months immunized with DPT | SDG 3 | Child immunization with DPT, measures the percentage of children aged 12 to 23 months who received DPT vaccines before 12 months or at any time before the survey. A child is considered to be adequately immunized against diphtheria, whooping cough and tetanus (DPT) after receiving three doses of the vaccine [10]. The average from 2010 to 2012 was calculated for each country. |
| Hospital beds per 1000 inhabitants’ rate | SDG 3 | Hospital beds include inpatient beds available in public, private, general and specialized hospitals and rehabilitation centers. In most cases, beds for acute and chronic care are included [11]. The average from 2010 to 2012 was calculated for each country. |
| Secondary life school expectancy, for both genders | SDG 4 | Number of years that a school-age person can expect to spend within the specified level of education. For a child of a certain age, school life expectancy is calculated as the sum of the age-specific enrollment rates for the specified education levels. The part of enrollment that is not distributed by age is divided by the school-age population by the level of education at which they are enrolled and multiplied by the duration of that level of education. The result is then added to the sum of the age-specific registration fees. A high school life expectancy indicates a greater likelihood that children will spend more years in education and greater overall retention within the education system. Note that the expected number of years does not necessarily coincide with the expected number of degrees completed, due to repetition. Since school life expectancy is an average based on participation at different levels of education, the expected number of years of schooling can be reduced by the magnitude of children who never attended school. Children in school can benefit from many more years of education than the average [12]. The average from 2010 to 2012 was calculated for each country. |
| Percentage of adolescents at high school age who are out of school | SDG 4 | Number of children of official high school age who did not attend primary or secondary school at any time during the reference school year, expressed as a percentage of the number of official children of secondary school age in the household survey sample. Children attending pre-primary or non-formal education are considered out of school. The UNESCO Institute for Statistics (UIS) releases estimates for out-of-school children calculated from administrative sources and household surveys (Demographic and Health Surveys and Multiple Indicator Cluster Surveys). Administrative records and household surveys are two sources of data that differ in fundamental ways: who collects the data and how, when and for what purpose. As a result, estimates of out-of-school children calculated from one data source may not correspond to those based on other data sources [13]. The average for the years 2010 to 2012 was calculated for each country. |
| Percentage of unemployed female labor force | SDG 5 | Female unemployment refers to the share of the labor force among women who are out of work, but available and seeking employment [69]. The average for the years 2010 to 2012 was calculated for each country. |
| Secondary school life expectancy among women | SDG 5 | Number of years a school-age girl can expect to spend within the specified level of education. For a girl of a certain age, school life expectancy is calculated as the sum of the age-specific enrollment rates for the specified levels of education. The part of enrollment that is not distributed by age is divided by the school-age population by the level of education at which they are enrolled and multiplied by the duration of that level of education. The result is then added to the sum of the age-specific registration fees. A high school life expectancy indicates that girls are more likely to spend more years in education and greater overall retention within the education system. Note that the expected number of years does not necessarily coincide with the expected number of degrees completed, due to repetition. Since school life expectancy is an average based on participation at different levels of education, the expected number of years of schooling can be reduced by the magnitude of girls who never attend school. Girls who are in school can benefit from many more years of education than the average [15]. The average from 2010 to 2012 was calculated for each country. |
| Proportion of seats held by women in national parliaments | SDG 5 | Women in parliaments are the percentage of parliamentary seats in a single chamber occupied by women [16]. The average from 2010 to 2012 was calculated for each country. |
| Percentage of people using basic sanitary facilities | SDG 6 | The percentage of people who use at least basic sanitation services, that is, improved sanitation facilities that are not shared with other families. This indicator covers both people who use basic sanitation services and those who use safely managed sanitation services. Improved sanitation facilities include discharge / discharge into sewer systems, septic tanks or pit latrines; improved ventilated pit latrines, composition toilets or pit latrines with slabs [17]. The average from 2010 to 2012 was calculated for each country. |
| Percentage of people using basic drinking water services | SDG 6 | The percentage of people who use at least basic water services. This indicator covers both people who use basic water services and those who use safely managed water services. Basic drinking water services are defined as drinking water from an improved source, as long as the collection time does not exceed 30 minutes for a round trip. Enhanced water sources include piped water, boreholes or pipes, protected dug wells, protected sources and packaged or delivered water.^18^ The average from 2010 to 2012 was calculated for each country. |
| Percentage of population with access to electricity | SDG 7 | Access to electricity is the percentage of the population with access to electricity. Electrification data are collected from industries, national surveys and international sources [19]. The average from 2010 to 2012 was calculated for each country. |
| Percentage of total unemployed workforce | SDG 8 | Unemployment refers to the portion of the labor force that is out of work, but available and looking for a job [20]. The average from 2010 to 2012 was calculated for each country. |
| Gross Domestic Product (GDP) per capita – Int$ PPP constant for 2017 | SDG 8 | Gross Domestic Product (GDP) per capita in Int$ PPP constant for 2017. |
| Inflation, consumer price | SDG 8 | Inflation measured by the consumer price index reflects the annual percentage change in the cost to the average consumer of purchasing a basket of goods and services that can be fixed or changed at specified intervals, such as annually. Laspeyres' formula is generally used [4]. The average for the years 2010 to 2012 was calculated for each country. |
| Gini Index | SDG 10 | The Gini index measures the extent to which the distribution of income (or, in some cases, consumption expenditure) between individuals or families in an economy deviates from a perfectly equal distribution. A Lorenz curve represents the cumulative percentages of total income received in relation to the cumulative number of recipients, starting with the poorest individual or household. The Gini index measures the area between the Lorenz curve and a hypothetical line of absolute equality, expressed as a percentage of the maximum area below the line. Thus, a Gini index of zero represents perfect equality, while an index of 100 implies perfect inequality [21]. The average of the years 2010 to 2012 was calculated for each country. |
| Percentage of urban population | SDG 11 | Urban population refers to people living in urban areas, as defined by national statistical offices [2]. The average for the years 2010 to 2012 was calculated for each country. |
| Corruption Control Index | SDG 16 | The control of corruption captures perceptions of the extent to which public power is exercised for private purposes, including small and large forms of corruption, as well as the cooptation of the State by elites and private interests. The estimate provides the country's score in the aggregate indicator, in units of a standard normal distribution, that is, ranging from approximately -2.50 to 2.50 [22]. The average of the years 2010 to 2012 was calculated for each country. |
| Government Effectiveness Index | SDG 16 | The government's effectiveness captures perceptions of the quality of public services and the degree of independence from political pressures, the quality of policy formulation and implementation, and the credibility of the government's commitment to these policies [23]. The average from 2010 to 2012 was calculated, for each country. |
| Political Stability and Absence of Violence/Terrorism Index | SDG 16 | Political stability and absence of violence / terrorism captures perceptions of the likelihood that the government will be destabilized or overthrown by unconstitutional or violent means, including politically motivated violence and terrorism [24]. The average from 2010 to 2012 was calculated for each country. |
| Regulatory Quality Index | SDG 16 | Regulatory quality captures perceptions of the government's ability to formulate and implement sound policies and regulations that allow and promote the development of the private sector [25]. The average from 2010 to 2012 was calculated for each country. |
| Rule of Law index | SDG 16 | The rule of law captures perceptions of the extent to which agents trust and comply with society's rules and the quality of contract enforcement, property rights, police and courts, as well as the likelihood of crime and violence [26]. Calculated the average for the years 2010 to 2012, for each country. |

Table S2 – Study variables distribution.

| **Variable** | **Observations** | **Mean** | **Standard**  **Deviation** | **Missing Observations** |
| --- | --- | --- | --- | --- |
| Neonatal mortality rate | 147 | 12.565 | 10.517 | 0 |
| Mortality rate among children aged 28 days or more and less than five years old | 147 | 15.827 | 19.083 | 0 |
| Public expenditure per capita – Int$ PPP constant for 2017 | 147 | 7058.136 | 8388.988 | 0 |
| Public expenditure in health per capita – Int$ PPP constant for 2017 | 147 | 888.365 | 1210.954 | 0 |
| Public expenditure in other sectors per capita – Int$ PPP constant for 2017 | 147 | 6169.771 | 7324.432 | 0 |
| Gross Domestic Product (GDP) per capita – Int$ PPP constant for 2017 | 147 | 16445.587 | 18010.858 | 0 |
| Population over 65 years old rate | 147 | 0.079 | 0.057 | 0 |
| Out-of-pocket disbursement with health per capita – Int$ PPP constant for 2017 | 147 | 266.556 | 263.233 | 0 |
| Gini Index | 102 | 29.360 | 6.079 | 45 |
| Income deficit of US$ 1.90 | 102 | 4.597 | 8.588 | 45 |
| Inflation, consumer price | 141 | 5.894 | 5.254 | 6 |
| Fertility rate | 147 | 2.964 | 1.523 | 0 |
| Percentage of total unemployed workforce | 145 | 7.894 | 5.887 | 2 |
| Percentage of people using basic sanitary facilities | 145 | 69.666 | 31.002 | 2 |
| Percentage of people using basic drinking water services | 144 | 82.959 | 18.813 | 3 |
| Percentage of unemployed female labor force | 145 | 9.013 | 6.982 | 2 |
| Secondary school life expectancy among women | 121 | 4.984 | 2.140 | 26 |
| Proportion of seats held by women in national parliaments | 144 | 19.684 | 10.892 | 3 |
| Secondary life school expectancy, for both genders | 122 | 5.030 | 2.017 | 25 |
| Percentage of adolescents at high school age who are out of school | 87 | 13.938 | 16.923 | 60 |
| Corruption Control Index | 145 | -0.182 | 0.997 | 2 |
| Government Effectiveness Index | 145 | -0.121 | 0.966 | 2 |
| Political Stability and Absence of Violence/Terrorism Index | 145 | -0.250 | 0.924 | 2 |
| Regulatory Quality Index | 145 | -0.062 | 0.962 | 2 |
| Rule of Law index | 145 | -0.200 | 0.988 | 2 |
| Malnutrition rate | 133 | 9.709 | 9.150 | 14 |
| Doctors per 1000 inhabitants’ rate | 119 | 1.846 | 1.578 | 28 |
| Births attended by a qualified health professional | 121 | 80.906 | 24.417 | 26 |
| HIV prevalence | 120 | 2.072 | 4.748 | 27 |
| Malaria incidence | 91 | 105.481 | 152.960 | 56 |
| Percentage of children between 12 and 23 months immunized with DPT | 145 | 88.195 | 12.603 | 2 |
| Hospital beds per 1000 inhabitants’ rate | 128 | 3.080 | 2.521 | 19 |
| Percentage of population with access to electricity | 144 | 76.221 | 32.339 | 3 |
| Percentage of urban population | 145 | 56.456 | 22.155 | 2 |
| Surface in km^2^ | 145 | 891773.383 | 2181091.265 | 2 |
| Population | 147 | 47053479.561 | 156292207.377 | 0 |
| Population density | 145 | 104.474 | 123.449 | 2 |
| Countries longitude | 147 | - | - | 0 |
| Country latitude | 147 | - | - | 0 |

Table S3 – Weights’ coefficients of variation.

|  | SuperLearner | Propensity Score | Covariate Balancing Propensity Score |
| --- | --- | --- | --- |
| Log of Mean Total Public Exp 2013-2017 | 1.578 | 0.701 | 0.399 |
| Log of Mean Health Public Exp 2013-2017 | 1.021 | 0.628 | 0.441 |
| Log of Mean Other Sec Public Exp 2013-2017 | 1.65 | 0.657 | 0.429 |

Legend: Log of Mean Total Public Exp 2013-2017: Natural logarithm of average public expenditure per capita of the years 2013 to 2017; Log of Mean Health Public Exp 2013-2017: Natural logarithm of average health public expenditure per capita of the years 2013 to 2017; Log of Mean Other Sec Public Exp 2013-2017: Natural logarithm of average public expenditure in other sectors than health per capita of the years 2013 to 2017.


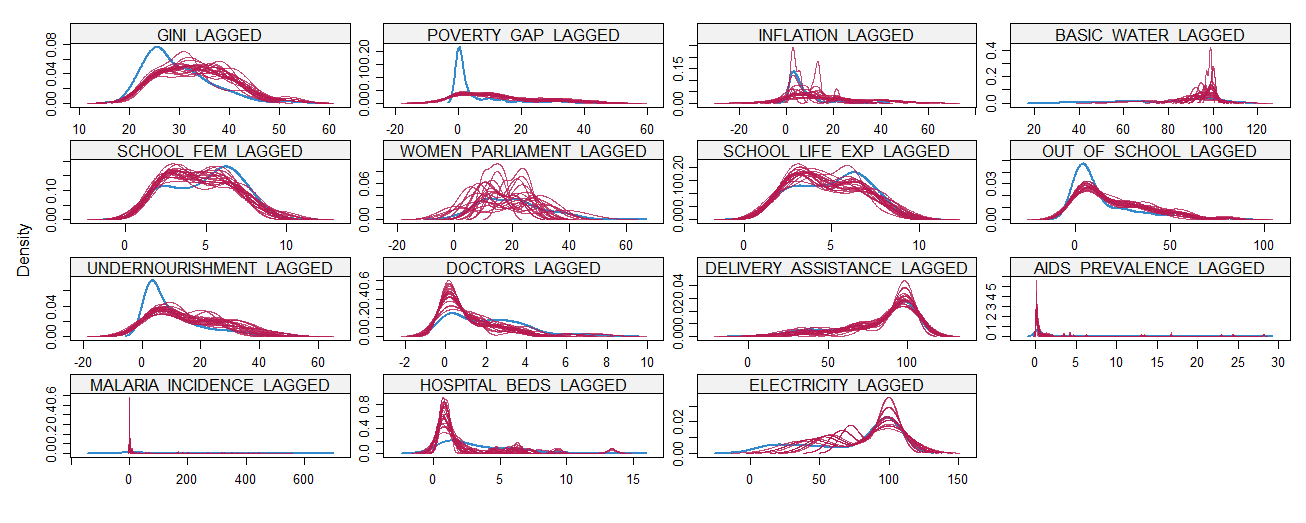


Figure S1: Density plots of the missing variables imputation.

Legend: The density of the imputed data for each data set is in red and that of the observed data in blue. GINI_LAGGED: Gini Index; POVERTY_GAP_LAGGED: Income deficit of US$ 1.90; INFLATION_LAGGED: Inflation, consumer price ; BASIC_WATER_LAGGED: Percentage of people using basic drinking water services; SCHOOL_FEM_LAGGED: Secondary school life expectancy among women; WOMEN_PARLIAMENT_LAGED: Proportion of seats held by women in national parliaments; SCHOOL_LIFE_EXP_LAGGED: Secondary life school expectancy, for both sexes; OUT_OF_SCHOOL_LAGGED: Percentage of adolescents at high school age who are out of school; UNDERNOURISHMENT_LAGGED: Malnutrition rate; DOCTORS_LAGGED: Doctors per 1000 inhabitants’ rate; DELIVERY_ASSISTANCE_LAGGED: Births attended by a qualified health professional; AIDS_PREVALENCE_LAGGED: HIV prevalence; MALARIA_INCIDENCE_LAGGED: Malaria incidence; HOSPITAL_BEDS_LAGGED: Hospital beds per 1000 inhabitants’ rate; ELECTRICITY_LAGGED: Percentage of population with access to electricity.
